# Perceived discrimination and mental health among adolescents in Germany

**DOI:** 10.1093/eurpub/ckac130.205

**Published:** 2022-10-25

**Authors:** M Blume, C Koschollek, K Kajikhina, J Waldhauer, C Hövener

**Affiliations:** Epidemiology and Health Monitoring, Robert Koch-Institut, Berlin, Germany

## Abstract

**Background:**

The association between perceived discrimination and mental health in adolescents has been widely documented. Aim of this contribution is to show how the relationship between mental health and perceived discrimination at school, work or job training differs depending on socio-economic or migration-related determinants.

**Methods:**

The present analyses was conducted with data from German Health Interview and Examination Survey for Children and Adolescents (KiGGS) wave 2 (2014 - 2017). Weighted linear regression analyses were conducted to explore the association between perceived discrimination at school, work or job training and behavioral problems in adolescents (14-17 years). Also, we examined the extent to which the association is moderated by the parents’ income, education as well as the language spoken at home.

**Results:**

Of all young people, 25.5 % reported perceived discrimination at school, work or job training. Behavioral problems were reported more frequently in adolescents with discrimination experience (12.5 % vs. 3.23 %). Multivariate analyses showed that the coefficients of the association between perceived discrimination and behavioral problems differed by parents’ level of education (low 4.07 (2.39-5.74)/high 2.47 (1.29-3.65)), income (low 3.61 (2.06-5.16)/high 0.35 (-1.81-2.51)), or language spoken at home (German 2.02 (0.33-3.70)/ Other 3.48 (-0.27-7.23)).

**Conclusions:**

A large proportion of adolescents experienced discrimination, with this being reported more frequently among young people with behavioral problems. Parents’ income and education as well as the language spoken at home are relevant for this context. To ensure that health inequalities are not exacerbated by discrimination, targeted prevention measures are needed in these settings. These should address not only the individual needs of young people, but also the underlying conditions and thus aim to promote health equity in the long term, especially in already disadvantaged groups.

**Key messages:**

- Perceived discrimination is important when considering mental health, whereby these associations may vary between socioeconomic and migration-related determinants.
- Prevention programs regarding discrimination are needed, which address individual needs and underlying conditions of adolescents, especially in already disadvantaged groups.

